# A fully coupled system of generalized thermoelastic theory for semiconductor medium

**DOI:** 10.1038/s41598-024-63554-2

**Published:** 2024-06-16

**Authors:** H. Sherief, M. Naim Anwar, A. Abd El-Latief, M. Fayik, A. M. Tawfik

**Affiliations:** 1 Department of Mathematics and Computer Sciences, Faculty of Sciences, Alexandria University, Alexandria, Egypt; 2 Department of Basic Sciences, Faculty of Engineering, Pharos University, Alexandria, Egypt; 3 Department of Mathematics, Faculty of Education, Alexandria University, Alexandria, Egypt; 4 Faculty of Computer Science and Engineering, Alamein International University, New Alamein, Egypt

**Keywords:** Danilovskaya’s problem, Direct approach, Maxwell–Cattenueo–Vernotte (MCV), Numerical results, Semiconductor, Materials science, Mathematics and computing

## Abstract

This study presents a new mathematical framework for analyzing the behavior of semiconductor elastic materials subjected to an external magnetic field. The framework encompasses the interaction between plasma, thermal, and elastic waves. A novel, fully coupled mathematical model that describes the plasma thermoelastic behavior of semiconductor materials is derived. Our new model is applied to obtain the solution to Danilovskaya’s problem, which is formed from an isotropic homogeneous semiconductor material. The Laplace transform is utilized to get the solution in the frequency domain using a direct approach. Numerical methods are employed to calculate the inverse Laplace transform, enabling the determination of the solution in the physical domain. Graphical representations are utilized to depict the numerical outcomes of many physical fields, including temperature, stress, displacement, chemical potential, carrier density, and current carrier distributions. These representations are generated for different values of time and depth of the semiconductor material. Ultimately, we receive a comparison between our model and several earlier fundamental models, which is then graphically represented.

## Introduction

In contemporary semiconductor technology, the shrinking size of devices poses challenges for thermal control. Consequently, it is imperative that we acquire a comprehensive comprehension of interconnected thermoelastic phenomena. The production of high-density integrated circuits (ICs) serves as a crucial illustration of this phenomenon. During the production process, specifically with methods like rapid thermal annealing (RTA), semiconductor wafers undergo sudden temperature fluctuations to activate dopants or rectify implantation damage. This process of thermal cycling results in significant temperature variations within the wafer. This results in substantial thermoelastic strains due to the mismatch in thermal expansion coefficients between the various layers of material. Applying these stresses can hurt the wafer in many ways, such as by warping it, making tiny cracks in it, and even causing major failures like delamination where the semiconductor meets other materials, like dielectrics or metal interconnects^1–3^.

Plasma, thermal, and elastic models are becoming essential tools in semiconductor engineering, particularly for accurately estimating stress distributions. These models are extremely useful tools throughout the design phase, making it easier to create semiconductor devices that are more durable and dependable. Engineers may optimize manufacturing processes by integrating real-time temperature fluctuations and their resulting mechanical reactions into these models, allowing them to adjust process settings and minimize the likelihood of mechanical failures. This rigorous optimization approach not only guarantees the dependability and efficiency of the ultimate semiconductor products but also makes a substantial contribution to enhancing overall production effectiveness and output. As a result, the inclusion of thermoelastic models is critical for the advancement of semiconductor technology and its various applications.

Lord and Shulman^4^ introduced their concept of generalized thermoelasticity with a single relaxation time as an alternative to Boit's coupled thermoelasticity theory^5^. Sherief and Saleh^6,7^ developed the theory of thermoelastic diffusion. According to this theoretical framework, both mechanical and thermal waves travel at finite speeds, in contrast to the unlimited speeds observed in the coupled theory. References^8–13^ provide applications of the generalized thermoelastic theory, which is associated with a single relaxation time. Abd El-Latief et al.^14^ recently published two novel methods that use four random walks to solve the 1D thermal shock problem in a thermoelastic half space using surrogate-based global optimization.

Thermoelasticity can significantly affect the electrical properties of a material. The thermoelastic effect can change a semiconductor's carrier concentration based on temperature. This phenomenon can lead to changes in the material's electrical conductivity as well as adjustments in its optical features. Gordon et al.^15^ were the first to discover the photothermal approach, where they showed that a laser-based device could cause photothermal blooming, leading to the formation of the photothermal lens. Kreuzer^16^ demonstrated the efficacy of photoacoustic spectroscopy for precise analysis using laser light sources. There are two types of photothermal processes that happen in semiconductor materials. The thermoelastic (TE) mechanism uses thermal waves to create elastic vibrations, while the electronic deformation (ED) mechanism uses photo-excited free carriers to make periodic elastic deformation^17^. Comprehensive theoretical analyses^18^ have described the mechanisms through coupled plasma, thermal, and elastic wave equations.

Many scholars have studied the uncoupled system consisting of plasma, thermal, and elastic equations. Mandelis^19^ conducted a study that examined the phenomenon of coupled thermal and plasma waves, also known as "thermoelectronic" waves. McDonald and Wetsel^20^ investigated the thermal and acoustic properties of a non-electronic material. Stearns and Kino^21^, and Todorovic^22^ have examined the system of partially coupled plasma, thermal, and elastic wave equations. Todorovic analyzes the reasons for disregarding the relationship between these equations. Furthermore, Todorovic employs the modified Fourier law to deduce a hyperbolic equation that characterizes the process of heat conduction^23^. The equation accurately predicts the limited speed at which heat waves travel through semiconductors. Researchers have studied the interaction between plasma and elastic waves on a semiconductor plate, also known as plasmaelastic (PE) waves^24^. Additional research built upon the existing groundwork, including the work of Song et al.^25^, who examined problems related to the reflection of light in semiconductors using generalized thermoelastic and plasma models. Othman et al.^26^ utilized normal mode analysis to study photothermal waves, whereas Abbas and Aly^27^ found analytical solutions for waves generated by concentrated laser beams. Later studies by Abbas^28^ and Hobiny and Abbas^29^ looked at how plasma, thermal, and elastic waves interact with cylinder-shaped cavities in semiconductor materials. Youssef et al.^30^ have introduced the hyperbolic two-temperature model in generalized thermoelasticity. Laser pulses cause semiconductor thermoelastic waves, as studied by Taye et al.^31^. Abo-Dahab and Lotfy^32^ investigated the problem of two-temperature difficulties involving two-dimensional deformations in semiconductors under mechanical force. Lotfy et al.^33^ used fractional-order models to look into what happens when the thermal conductivity of semiconductors with cavities changes. Previous studies^31,34^ have investigated the impact of changing thermal conductivity on semiconductors. The study^35^ investigates how non-local theory influences photo-thermal wave transmission in semiconductors. It specifically focuses on using the two-phase lag theory of thermoelasticity and a modified Moore-Gibson-Thompson heat conduction equation. The impact of the Seebeck effect on flexible electrically conductive or semiconductive materials is examined in^36^.

Our work is divided into six primary sections. The first section aims to present a derivation for a novel and all-encompassing mathematical model that describes the thermoelastic properties of semiconductor materials. This model takes into account the impact of an external magnetic field and heat source, as well as the interactions between plasma, thermal, and elastic waves in all governing equations. It is distinct from previous models in this regard. In the second section, we utilize this model to address Danilovskaya's problem without any external force. We employ the Laplace transform to obtain responses in the frequency domain. Numerical methods are used in the third section to compute the inverse Laplace transform^37,38^, which yields solutions in the physical domain. Graphical representations are employed to depict several physical fields, including temperature, tension, displacement, chemical potential, carrier density, and current carrier distributions. The fourth section investigates the significance of the particle diffusion relaxation time and its influence on carrier current. The fifth section of the paper presents a comparison between the outcomes achieved using the proposed model and those acquired from earlier models, emphasizing the enhancements and disparities. Ultimately, we have incorporated the research paper's conclusions in the last section.

## Derivation of the fundamental equations

Let $V$ stands for any volume of semiconductor material that is enclosed by a surface $A$. Consequently, the rule of conservation of energy for $V$ can be expressed as^6^

1 $$ \begin{aligned}    & \frac{d}{{dt}}\left( {\int _{V} \frac{1}{2}\rho \dot{u}_{i} \dot{u}_{i} dV + \int _{V} \rho UdV} \right) \\     & \quad  = \int _{V} \rho F_{i} \dot{u}_{i} dV + \int _{V} M\dot{N}dV + \int _{A} \sigma _{{ij}} \dot{u}_{i} n_{j} dA - \int _{A} q_{j} n_{j} dA + \int _{V} \rho QdV, \\  \end{aligned} $$

where $N=n-{n}_{0}$, and $M$ is the energy added to the system when adding one particle without adding heat or work^39^. The presence of superimposed dots signifies time derivatives, and the summation convention rule is applied as usual.

The equation of motion and the symmetry of the stress tensor are given by

2 $${\sigma }_{ji,j}+\rho {F}_{i}={\rho \ddot{u}}_{i}, {\sigma }_{ij}={\sigma }_{ji}.$$

Apply Gauss’ divergence theorem to Eq. (1), and using Eq. (2), we get the pointwise form

3 $$\rho \dot{U}={\sigma }_{ij}{\dot{e}}_{ij}-{q}_{i,i}+\rho Q+M\dot{N},$$

The entropy equation is given by^40,41^

4 $$\rho T\dot{S}=-{q}_{i,i}+\rho Q,$$

Equation (3) after using Eq. (4), becomes

5 $$\rho dU={\sigma }_{ij}{de}_{ij}+\rho TdS+MdN.$$

We now define the Helmholtz free energy function $\psi$ as

6 $$\psi =U-TS.$$

Eliminating *U* between Eq. (5) and Eq. (6), we get

7 $$\rho d\psi ={\sigma }_{ij}d{e}_{ij}+MdN-\rho SdT.$$

The function $\psi$ (as well as all other considered functions) may be defined in terms of the independent variables ${e}_{ij}$, $T,$ and *N*, then by using the chain rule, we obtain

8 $$d\psi =\frac{\partial \psi }{\partial {e}_{ij}}d{e}_{ij}+\frac{\partial \psi }{\partial N}dN+\frac{\partial \psi }{\partial T}dT.$$

Comparing both Eqs. (7) and (8), yields

9 $${\sigma }_{ij}=\rho \frac{\partial \psi }{\partial {e}_{ij}},$$

10 $$S=-\frac{\partial \psi }{\partial T},$$

11 $$M=\rho \frac{\partial \psi }{\partial N}.$$

The function $\psi$ is now expanded as a power series of the mentioned independent variables in the given form^42^

12 $$\rho \psi =\rho {\psi }_{0}+{a}_{0}N+{b}_{0}\theta +{{c}_{ij} e}_{ij}-\frac{\rho {C}_{E}}{2{T}_{0}}{\theta }^{2}+\frac{1}{2}{bN}^{2}+\frac{1}{2}{{C}_{ijkl} e}_{ij}{e}_{kl}+{b}_{ij}{e}_{ij}N+{a}_{ij}{e}_{ij}\theta +aN\theta +\dots,$$

where $\theta =T-{T}_{0}$, and $\left|\frac{\theta }{{T}_{0}}\right|\ll 1$. Both $a$ and $b$ are physical constants with dimensions [$J/K$] and [$J.{m}^{3}$].

Now, in the medium's natural state, we have

13 $$\psi =0, { e}_{ij}=0, N=0, \theta =0\, and\, {\sigma }_{ij}=0.$$

Hence,

14 $${\psi }_{0}=0, { {{c}_{ij}=0, a}_{0}=0 \,and \,b}_{0}=0.$$

Therefore, we can rewrite Eq. (12) as follows (ignoring terms of higher order):

15 $$\rho \psi =\frac{-\rho {C}_{E}}{2{T}_{0}}{\theta }^{2}+{b}_{ij}{e}_{ij}N+\frac{1}{2}{{C}_{ijkl} e}_{ij}{e}_{kl}+{a}_{ij}{e}_{ij}\theta +aN\theta +\frac{1}{2}{bN}^{2}.$$

Using Eqs. (9)–(10) into Eq. (15), we obtain

16 $${\sigma }_{ij}={C}_{ijkl} {e}_{kl}+{a}_{ij}\theta +{b}_{ij}N,$$

17 $$M={b}_{ij} {e}_{ij}+a\theta +bN,$$

18 $$\rho S=\frac{\rho {C}_{E}}{{T}_{0}}\theta -{a}_{ij}{e}_{ij}-aN.$$

For the isotropic case, we have the following relations.

19 $${C}_{ijkl}=\lambda {\delta }_{ij}{\delta }_{kl}+\mu {\delta }_{ik}{\delta }_{jl}+\mu {\delta }_{il}{\delta }_{jk},$$

20 $${a}_{ij}=-{\beta }_{1}{\delta }_{ij}=-\left(3\lambda +2\mu \right){\alpha }_{t}{\delta }_{ij},$$

21 $${b}_{ij}=-{\beta }_{2}{\delta }_{ij}=-\left(3\lambda +2\mu \right){\alpha }_{n}{\delta }_{ij},$$

where ${\alpha }_{n}$ represents the discrepancy in deformation potential between the conduction and valence bands^22^.

Furthermore, the parameters $a$ and $b$ can be written as follows:^23^

22 $$a=\frac{{E}_{g}}{{T}_{0}}, b=\frac{{q}_{0}^{2}{D}_{e}}{{\sigma }_{0} }.$$

By substituting Eqs. (19)–(20) into Eqs. (16)–(17), we obtain their form in the isotropic case.

23 $${\sigma }_{ij}=2\mu {e}_{ij}+{\delta }_{ij}(\lambda {e}_{kk}-{\beta }_{1}\theta -{\beta }_{2}N),$$

24 $$M=\frac{{E}_{g}}{{T}_{0}}\theta - {\beta }_{2}{e}_{kk}+bN,$$

25 $$\rho S{T}_{0}=\rho {C}_{E}\theta +{T}_{0}{\beta }_{1}{e}_{kk}-aN{T}_{0}.$$

By substituting Eq. (23) into Eq. (2) with the definition of the strain tensor ${e}_{ij}=\frac{1}{2}({u}_{i,j}+{u}_{j,i})$, we get

26 $$\mu {u}_{i,jj}+\left(\lambda +\mu \right){u}_{j,ij}-{\beta }_{1}{\theta ,}_{i}-{\beta }_{2}{N,}_{i}+\rho {F}_{i}={\rho \ddot{u}}_{i}.$$

We can rewrite Eq. (4) in a linearized form as follows:

27 $${q}_{i,i}=-\rho {T}_{0}\dot{S}+\rho Q.$$

Using the above equation into Eq. (25), we obtain

28 $${q}_{i,i}=-\rho {C}_{E}\dot{\theta }-{\beta }_{1}{T}_{0}{\dot{e}}_{kk}+a{T}_{0}\dot{N}+\rho Q.$$

We now assume the generalized Fourier law of heat conduction as

29 $${q}_{i}+{{\tau }_{0}\dot{q}}_{i}=-k{\theta }_{,i},$$

Next, we apply the divergence operator to both sides of Eq. (29) and use Eq. (28) along with its time derivative to arrive at the equation of heat conduction.

30 $$\rho {C}_{E}\left(\dot{\theta }+{\tau }_{0}\ddot{\theta }\right)+{\beta }_{1}{T}_{0}\left(\dot{e}+{\tau }_{0}\ddot{e}\right)-{E}_{g}\left(\dot{N}+{\tau }_{0}\ddot{N}\right)-\rho \left(Q+{\tau }_{0}\dot{Q}\right)=k{\theta }_{,ii}.$$

For low electric field, the current carrier density vector $\mathbf{J}$ is given by^43^

31 $$\mathbf{J}={\sigma }_{0}\left(\mathbf{E}+\dot{\mathbf{u}}\wedge \mathbf{B}\right),$$

where the bold letters refer to vector quantities, and the magnetic induction $\mathbf{B}$ is given by the relation

32 $$\mathbf{B}={\mu }_{0}\left({\mathbf{H}}_{0}+\mathbf{h}\right),$$

Equation (31) can be rewritten for a semi-conductor medium in terms of chemical potential as follows^44^

33 $$\mathbf{J}={\sigma }_{0}\left(\frac{-\nabla M}{{q}_{0}}+\dot{\mathbf{u}}\wedge \mathbf{B}\right).$$

We postulate an analogous equation for the current density vector $\mathbf{J}$, similar to Eq. (29) for the heat flux vector

34 $$\mathbf{J}+{\tau }_{1}\dot{\mathbf{J}}={\sigma }_{0}\left(\frac{-\nabla M}{{q}_{0}}+\dot{\mathbf{u}}\wedge \mathbf{B}\right).$$

The significance of the aforementioned modification will be addressed in section "Particle diffusion relaxation time : significance and impact on current carrier" at a later point.

The conservation of charge equation for semiconductors is^45^

35 $$\nabla \cdot \mathbf{J}=-{q}_{0}\left(\dot{N}+\frac{N}{{\tau }_{R}}-G\right).$$

By applying the divergence operator to both sides of Eq. (34) and utilizing Eq. (24) and Eq. (35), we obtain

36 $$\left(1+{\tau }_{1}\frac{\partial }{\partial t} \right)\left(\dot{N}+\frac{1}{{\tau }_{R}}N-G \right)=\frac{{{E}_{g}\sigma }_{0}}{{q}_{0}^{2}{T}_{0}}{\nabla }^{2}\theta -\frac{{{\beta }_{2}\sigma }_{0}}{{q}_{0}^{2}} {\nabla }^{2}e+\frac{{b\sigma }_{0}}{{q}_{0}^{2}}{\nabla }^{2}N-\frac{{\sigma }_{0}}{{q}_{0}}\left(\nabla \cdot \left(\dot{\mathbf{u}}\wedge \mathbf{B}\right)\right).$$

Therefore, our model is formulated, comprising three equations: the equation of motion (Eq. (26)), the equation of heat conduction (Eq. (30)), and the particle diffusion equation (Eq. (36)), which are supplemented by the constitutive equations (Eqs. (23)-(24)). The body force term in the equation of motion can be attributed to the Lorentz force, which is expressed as.

37 $$\mathbf{F}=\mathbf{J}\wedge \mathbf{B}$$

Lorentz's force explains the mathematical equations and the physical significance of the forces exerted on charged particles when they traverse through a region of space that contains both an electric field and a magnetic field.

## Formulation of the problem

Consider a homogeneous isotropic semiconductor material occupying the region of the half space $0\le x<\infty$ that is taken to be traction free and is subjected to a time dependent thermal shock where the temperature is assumed to be a known function of time on the surface of the half plane. So, due to the physics of the problem, all the functions will depend on $x$ and $t$ only; therefore, the displacement components become ${u}_{x}=u\left(x,t\right)$, ${u}_{y}={u}_{z}=0$.

In the absence of body force, heat source, carrier source, and external applied magnetic field. The governing equations can be expressed as

38 $$\mu \frac{{\partial }^{2}u}{\partial {x}^{2}}+\left(\lambda +\mu \right)\frac{\partial e}{\partial x}-{\beta }_{1}\frac{\partial \theta }{\partial x}-{\beta }_{2}\frac{\partial N}{\partial x}={\rho \ddot{u}},$$

39 $$\rho {C}_{E}\left(\dot{\theta }+{\tau }_{0}\ddot{\theta }\right)+{\beta }_{1}{T}_{0}\left({\dot{e}}_{ij}+{\tau }_{0}\ddot{{e}_{ij}}\right)-{E}_{g}\left(\dot{N}+{\tau }_{0}\ddot{N}\right)=k\frac{{\partial }^{2}\theta }{\partial {x}^{2}},$$

40 $$\left(1+{\tau }_{1}\frac{\partial }{\partial t} \right)\left(\dot{N}+\frac{1}{{\tau }_{R}}N \right)=\frac{{\sigma }_{0}{E}_{g}}{{q}_{0}^{2}{T}_{0}}\frac{{\partial }^{2}\theta }{\partial {x}^{2}}-\frac{{\sigma }_{0}{\beta }_{2}}{{q}_{0}^{2}} \frac{{\partial }^{2}e}{\partial {x}^{2}}+\frac{{\sigma }_{0}b}{{q}_{0}^{2}}\frac{{\partial }^{2}N}{\partial {x}^{2}}.$$

In addition to the constitutive equations

41 $${\sigma }_{xx}=(2\mu {+\lambda )e}_{kk}-{\beta }_{1}\theta -{\beta }_{2}N,$$

42 $$M=\frac{{E}_{g}}{{T}_{0}}\theta - {\beta }_{2}{e}_{kk}+bN.$$

The non-dimensional variable is given by the following relations $\left( {x,u,t} \right) = \frac{1}{{c_{1} \eta }}\left( {x^{*} ,u^{*} ,\frac{1}{{c_{1} }}t^{*} } \right),(\theta ,N) = \frac{\lambda + 2\mu }{{\beta_{1} \beta_{2} }}\left( {\beta_{2} \theta^{*} ,\beta_{1} N^{*} } \right),\sigma_{ij} = \left( {\lambda + 2\mu } \right)\sigma_{ij}^{*} ,$

43 $$M={\beta }_{2}{M}^{*},$$

where $${c}_{1}^{2}=\frac{\lambda +2\mu }{\rho }$$ , $$\eta =\frac{\rho {C}_{E}}{k}.$$

Equations (38)–(39) in the dimensionless form become

44 $$\frac{{\partial }^{2}u}{\partial {x}^{2}}+\left({\beta }^{2}-1\right)\frac{\partial e}{\partial x}-{\beta }^{2}\frac{\partial \theta }{\partial x}-{\beta }^{2}\frac{\partial N}{\partial x}={\beta }^{2}\ddot{u},$$

45 $$\left(\frac{\partial }{\partial t}+{\tau }_{0 }\frac{{\partial }^{2}}{\partial {t}^{2}}\right) \left(\theta +\varepsilon e-{\alpha }_{1}N\right)=\frac{{\partial }^{2}\theta }{\partial {x}^{2}},$$

46 $${\alpha }_{2}\left(1+{\tau }_{1}\frac{\partial }{\partial \text{t}} \right)\left(\dot{N}+\frac{1}{{\tau }_{R}}N \right)={\alpha }_{3}\frac{{\partial }^{2}\theta }{\partial {x}^{2}}- {\alpha }_{4}\frac{{\partial }^{2}e}{\partial {x}^{2}}+{\alpha }_{5}\frac{{\partial }^{2}N}{\partial {x}^{2}},$$

47 $${\sigma }_{xx}=e-\theta -N,$$

48 $$M={\alpha }_{6}\theta - e+{\alpha }_{7}N,$$

where

$$\beta^{2} = \frac{\lambda + 2\mu }{\mu }, \varepsilon = \frac{{\beta_{1}^{2} T_{0} }}{{\left( {\lambda + 2\mu } \right)\rho C_{E} }},\alpha_{1} = \frac{{E_{g} \beta_{1} }}{{\rho C_{E} \beta_{2} }},\alpha_{2} = \frac{{q_{0}^{2} \left( {\lambda + 2\mu } \right)}}{{\eta \beta_{2} }},\alpha_{3} = \frac{{\left( {\lambda + 2\mu } \right)\sigma_{0} E_{g} }}{{\beta_{1} T_{0} }},\alpha_{4} = \sigma_{0} \beta_{2},$$

$$\alpha_{5} = \frac{{\sigma_{0} b\left( {\lambda + 2\mu } \right)}}{{\beta_{2} }},\alpha_{6} = \frac{{E_{g} \left( {\lambda + 2\mu } \right)}}{{\beta_{1} \beta_{2} T_{0} }},\alpha_{7} = \frac{{b\left( {\lambda + 2\mu } \right)}}{{\beta_{2}^{2} }}.$$

The initial conditions of the problem are taken to be homogeneous w

49 $${\left.\theta (x,t)\right|}_{x=0}=H(t) ,{\left. \theta \left(x,t\right)\right|}_{x=\infty }=0,$$

hile the boundary conditions are assumed to be

50 $${\left.{\sigma }_{xx}(x,t)\right|}_{x=0}=0 ,{\left. \sigma (x,t)\right|}_{x=\infty }=0,$$

51 $${\left.\frac{dN(x,t)}{dx}\right|}_{x=0}=0 , {\left. N(x,t)\right|}_{x=\infty }=0,$$

where $H(t)$ is the Heaviside unit step function defined by

52 $$H\left(t\right)=\left\{\begin{array}{c}1\quad if \quad t\ge 0\\ 0 \quad otherwise.\end{array}\right.$$

## Solution in the Laplace transform domain

We now define the Laplace transform for a function $f\left(t\right)$ by the relation

53 $$\overline{f }\left(s\right)={\int }_{0}^{\infty }{e}^{-st}f\left(t\right) dt.$$

Applying Laplace transform on Eqs. (44)–(45), and using the homogeneous initial conditions, we get

54 $${(D}^{2}-{s}^{2})\overline{e }-{D}^{2}\overline{\theta }-{D}^{2}\overline{N }=0,$$

55 $$\left(s+{\tau }_{0 }{s}^{2}-{D}^{2}\right)\overline{\theta }+\varepsilon s\left(1+{\tau }_{0 }s\right)\overline{e }-{\alpha }_{1}s\left(1+{\tau }_{0}s\right)\overline{N }=0,$$

56 $${\alpha }_{4}{D}^{2}\overline{e }-{{\alpha }_{3}D}^{2}\overline{\theta }+\left[{\alpha }_{2}\left({\tau }_{1}s+1\right)\left(s+\frac{1}{{\tau }_{R}} \right)-{\alpha }_{5}{D}^{2}\right]\overline{N } =0,$$

57 $${\overline{\sigma }}_{xx}={\overline{e }}-{\overline{\theta }}-{\overline{N }},$$

58 $$\overline{M }={\alpha }_{6}\overline{\theta }- \overline{e }+{\alpha }_{7}\overline{N },$$

where $$D=\frac{d}{dx}$$.

By simultaneously solving Eqs. (54)–(55), we obtain the following differential equation.

59 $$\left({D}^{6}-{\xi }_{1}{D}^{4}+{\xi }_{2}{D}^{2}-{\xi }_{3}\right) \left\{\begin{array}{c}\overline{\theta }(x,s)\\ \overline{e }(x,s)\\ \overline{N }(x,s)\end{array}\right. =0,$$

where

$${\xi }_{1}=\left[\left({\alpha }_{1}\left({\alpha }_{3}-{\alpha }_{4}\right)-\varepsilon {\alpha }_{3}-{\alpha }_{4}\right)s{l}_{0}+{\alpha }_{5}\left(\left(\varepsilon +1\right)s{l}_{0}+{s}^{2}\right)+{l}_{0}\right]/\left({\alpha }_{5}-{\alpha }_{4}\right),$$

$${\xi }_{2}=\left[\left({\alpha }_{1}{\alpha }_{3}+{\alpha }_{5}\right){s}^{3}{l}_{0}+\left(\varepsilon +1\right)s{l}_{0}{l}_{1}+{l}_{1}{s}^{2}\right]/\left({\alpha }_{5}-{\alpha }_{4}\right),$$

$${\xi }_{3}=\left({s}^{3}{l}_{0}{l}_{1}\right)/\left({\alpha }_{5}-{\alpha }_{4}\right), and \,{l}_{0}=1+{\tau }_{0 }s, {l}_{1}={\alpha }_{2}\left({1+\tau }_{1 }s\right)\left(s+\frac{1}{{\tau }_{R}} \right).$$

The bounded solutions of Eq. (59) as $x\to \infty$ are given by

60 $$\overline{\theta }=\sum_{i=1}^{3}{A}_{i}\left(s\right){e}^{-{k}_{i}x},$$

61 $$\overline{e }=\sum_{i=1}^{3}{B}_{i}(s){e}^{-{k}_{i}x},$$

62 $$\overline{N }={\sum }_{i=1}^{3}{D}_{i}(s){e}^{-{k}_{i}x},$$

where ${A}_{i}\left(s\right), {B}_{i}\left(s\right)$ and ${D}_{i}(s)$ are parameters to be determined and ${k}_{1},{k}_{2}$ and ${k}_{3}$ are the roots with positive real part of the following characteristic equation

63 $${k}^{6}-{\xi }_{1}{k}^{4}+{\xi }_{2}{k}^{2}-{\xi }_{3}=0.$$

The roots *k*_1_, *k*_2_, and *k*_3_ of the above characteristic equation are given by

$${k}_{1}=\sqrt{\frac{1}{3}\left(2p\text{sin}\left(q\right)+{\xi }_{1}\right)},$$

$${k}_{2}=\sqrt{\frac{1}{3}\left({\xi }_{1}-p\left(\sqrt{3}\text{cos}\left(q\right)+\text{sin}(q)\right)\right),}$$

$${k}_{3}=\sqrt{\frac{1}{3}\left({\xi }_{1}+p\left(\sqrt{3}\text{cos}\left(q\right)-\text{sin}(q)\right)\right)},$$

where $$p=\sqrt{{\xi }_{1}^{2}-3{\xi }_{2}}$$ , $$q=\frac{1}{3}{\text{sin}}^{-1}(r)$$ and $$r=-\frac{2{\xi }_{1}^{3}-9{\xi }_{1}{\xi }_{2}+27{\xi }_{3}}{2{p}^{3}}$$.

By substituting Eqs. (60)–(61) into Eqs. (44)–(45), after some manipulations, we obtain

64 $$\overline{\theta }=\sum_{i=1}^{3}{\varphi }_{i}(s){C}_{i}{e}^{-{k}_{i}x},$$

65 $$\overline{e }=\sum_{i=1}^{3}{\psi }_{i}(s){{k}_{i}}^{2}{C}_{i}{e}^{-{k}_{i}x},$$

66 $$\overline{N }={\sum }_{i=1}^{3}{\left[\left({{k}_{i}}^{2}-{s}^{2}\right){\psi }_{i}\left(s\right)-{\varphi }_{i}(s)\right]C}_{i}{e}^{-{k}_{i}x},$$

67 $${\overline{\sigma }}_{xx}={s}^{2}\sum_{i=1}^{3}{\psi }_{i}\left(s\right){C}_{i}{e}^{-{k}_{i}x},$$

68 $$\overline{M }=\sum_{i=1}^{3}{\left[{\left({{\alpha }_{6}-\alpha }_{7}\right)\varphi }_{i}\left(s\right)+\left(\left({\alpha }_{7}-1\right){{k}_{i}}^{2}-{\alpha }_{7}{s}^{2}\right){\psi }_{i}\left(s\right)\right]C}_{i}{e}^{-{k}_{i}x},$$

where $${\phi }_{i}\left(s\right)=s{l}_{0}\left[\left({\alpha }_{1}-\varepsilon \right){{k}_{i}}^{2}-{\alpha }_{1}{s}^{2}\right], {\psi }_{i}\left(s\right)=\left(s{l}_{0}\left(1+{\alpha }_{1}\right)-{{k}_{i}}^{2}\right).$$

## Numerical result and discussion

In order to present numerical results in an effective way, a Silicon material was chosen for purposes of numerical evaluation, whose parameters in SI units are listed in Table 1^46–50^.

**Table 1:** Physical material parameters of silicon in SI units at ${T}_{0}=293K$.

Physical material parameters	Values	Units
$\rho$	$2330$	${\text{Kgm}}^{-3}$
$\lambda$	$3.64\times {10}^{10}$	${\text{Nm}}^{-2}$
$\mu$	$5.46\times {10}^{10}$	${\text{Nm}}^{-2}$
${C}_{E}$	$695$	${\text{JKg}}^{-1}{\text{K}}^{-1}$
$k$	$150$	${\text{Wm}}^{-1}{\text{K}}^{-1}$
${\alpha }_{t}$	$3\times {10}^{-6}$	${\text{K}}^{-1}$
${\alpha }_{n}$	$-9\times {10}^{-31}$	${\text{m}}^{3}$
$b$	$3.82\times {10}^{-39}$	${\text{Jm}}^{3}$
${E}_{g}$	$1.11 \times {10}^{-19}$	$\text{eV}$
${D}_{e}$	$2.5\times {10}^{-3}$	${\text{m}}^{2}{\text{s}}^{-1}$
${\sigma }_{0}$	$1.67\times {10}^{-2}$	${\Omega }^{-1}{\text{cm}}^{-1}$
${q}_{0}$	$1.6\times {10}^{-19}$	$\text{C}$
${\tau }_{0}$	$3\times {10}^{-14}$	$\text{sec}$
${\tau }_{1}$	$3\times {10}^{-13}$	$\text{sec}$
${\tau }_{r}$	$1.5{\times 10}^{-14}$	$\text{sec}$

In order to invert the Laplace transform in the above Eqs. (64)–(65), we adopt a numerical inversion method based on a Fourier series expansion^51^. The numerical inversion method used to find the solution in the physical domain is listed in^52–58^. The FORTRAN programming language generated the numerical code. The MATLAB software, with its powerful graphical capabilities, visually represented the numerical results, maintaining a precision of ten digits in the numerical algorithm.

**Figure 1:**
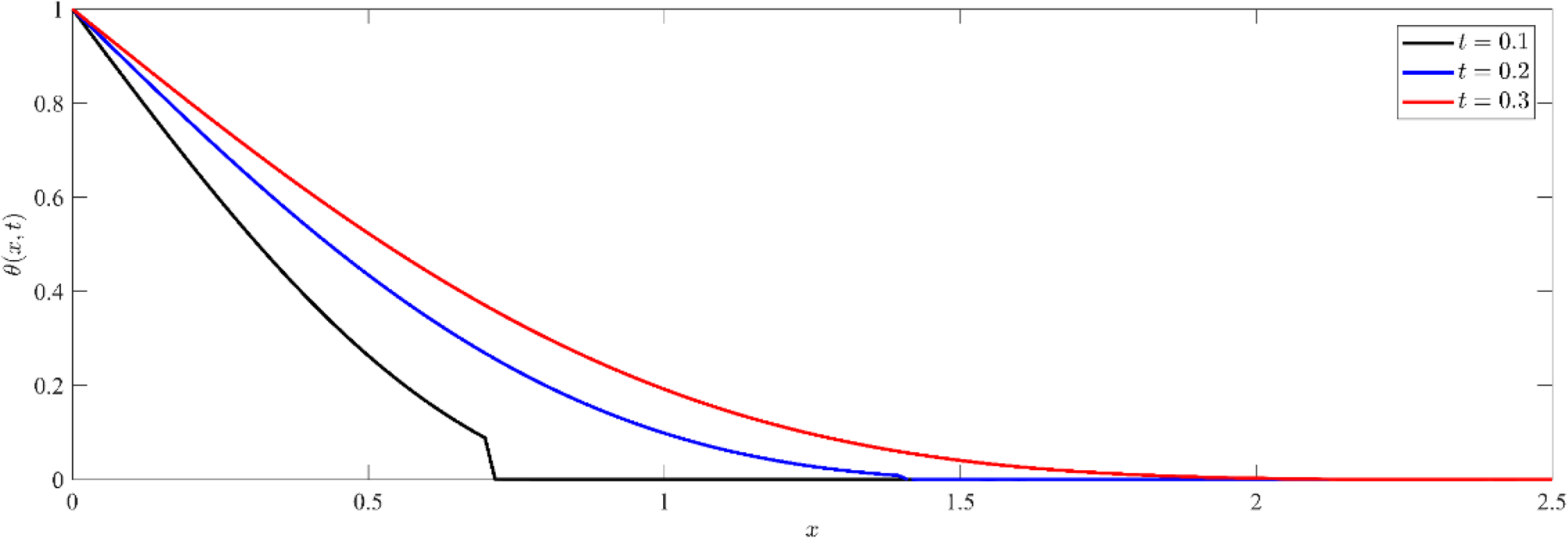
Non dimensional temperature distribution $\theta (x,t)$ at different values of time.

**Figure 2:**
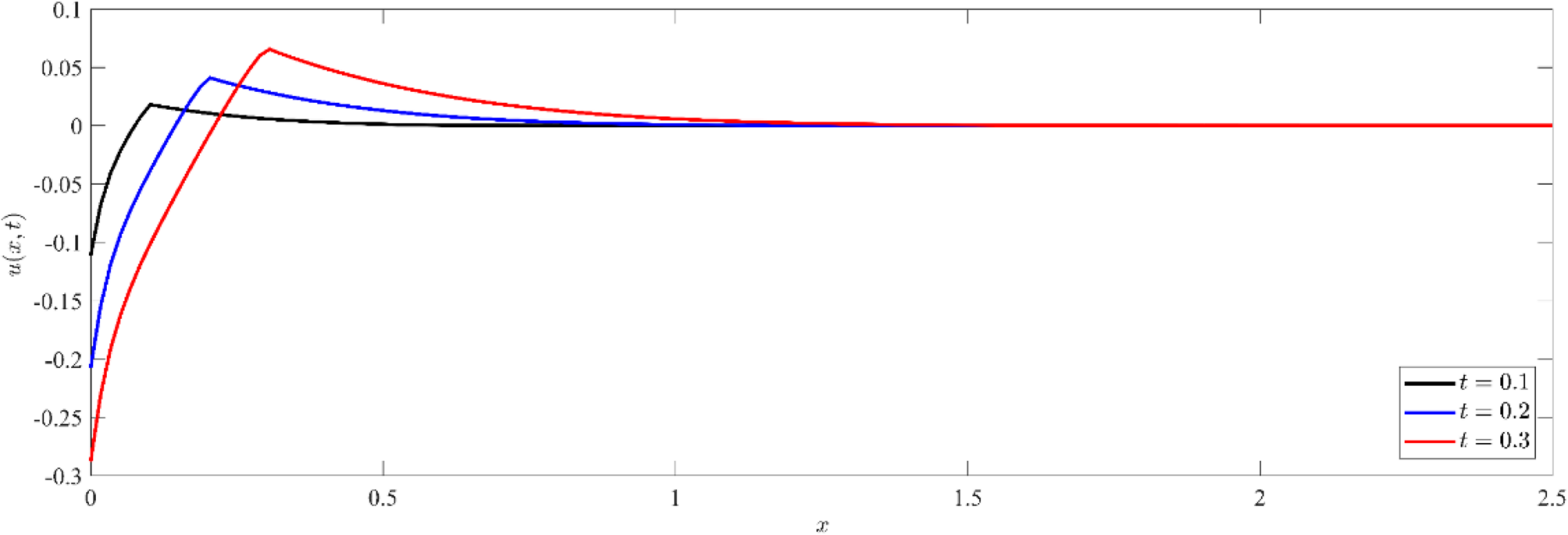
Non dimensional displacement distribution $u(x,t)$ at different values of time.

**Figure 3:**
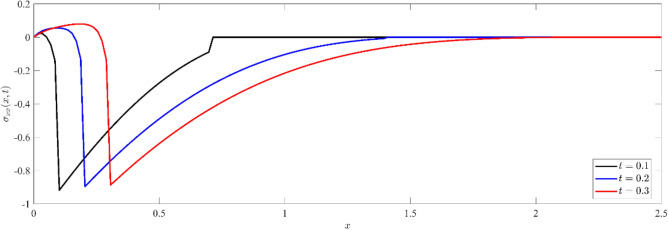
Non dimensional normal stress distribution ${\sigma }_{xx}(x,t)$ at different values of time.

Figures 1, 2, 3 show that temperature, displacement, and stress act in line with the generalized theory of thermoelasticity, which predicts a finite speed for small times, and behave like the coupled theory, which predicts an infinite speed for large times.

Figure 1 is plotted to show the variation of dimensionless temperature $$\theta (x,t)$$ against $$x-$$axis for three different instants of time, namely $$t=0.1, 0.2$$ and $$t=0.3$$. We observe that at any fixed time, the temperature distribution reaches its maximum value at the boundary, aligning with the thermal boundary condition that subjects the half space's boundary surface to a thermal shock. Inside the medium, the values slowly decay. For small values of time $$(t=0.10, say)$$, the values drop to zero, and the sharp temperature decreases to zero due to the effect of the thermal wave. This behavior is consistent with the generalized theory of thermoelasticity, which predicts finite wave speeds for short durations. Over longer values of time ($$t=0.2 or 0.3)$$, the temperature profile aligns with the coupled theory, exhibiting characteristics of infinite speed propagation. This shift indicates that initially, thermal effects are localized, but over longer times, the effects spread rapidly throughout the material.

Figure 2 depicts the variation of dimensionless displacement $u(x,t)$ against $x-$ axis for three different instants of time, namely $t=0.1, 0.2$ and $t=0.3$. We notice that for any fixed value of time, the displacement records a negative value at the boundary. This initial value indicates a significant mechanical response to the thermal input. The displacement value then increases with distance, reaching its maximum positive value, before gradually decreasing to zero. In addition, when the values of time increase, the magnitude of the displacement increases on the whole domain and cut $x$-axis more lately, signifying an increase in the rate of mechanical deformation. We plot Fig. 3 to illustrate the variation of the dimensionless stress component ${\sigma }_{xx}(x,t)$ with distance $x$ at various value of time. Initially, the stress distribution shows a sharp gradient near the surface, indicating significant stress generation due to thermal effects. For any fixed value of time, the magnitude of stress increases from zero values at the boundary to the maximum values, then decreases with the increase in distance, and finally diminishes to zero. This two-way interaction between the thermal and mechanical waves in the non-dimensional temperature distribution and non-dimensional stress distribution shows how complicated it is for silicon's thermal and mechanical responses to interact with each other. Also, for small values of time, the locations of the thermal and mechanical wave fronts coincide in Figs. 1 and 3. This is evidence of the accuracy of the numerical calculations provided.

**Figure 4:**
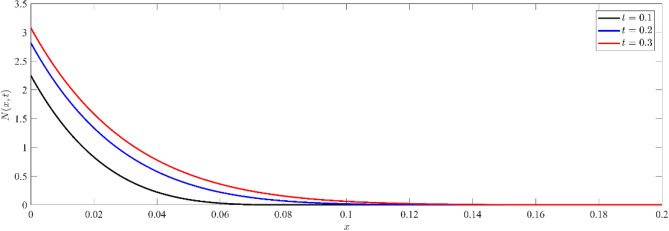
Non dimensional carrier density distribution $N(x,t)$ at different values of time.

Figure 4 illustrates the non-dimensional distribution of the carrier density $N(x,t)$ against $x$-axis for three different instants of time, namely $t=0.1, 0.2$ and $t=0.3$. At the boundary $x=0$, there is a high concentration of particles near the surface, which decreases as they diffuse deeper into the silicon medium. Raising the temperature leads to an augmentation in the movement of electrons from the valence band to the conduction band, resulting in an improvement in conductivity and a decrease in resistance. This behavior is in line with the principles of semiconductor physics, where an increase in temperature has a substantial impact on the number of free charge carriers. This phenomenon demonstrates the impact of temperature on the movement of carriers.

**Figure 5:**
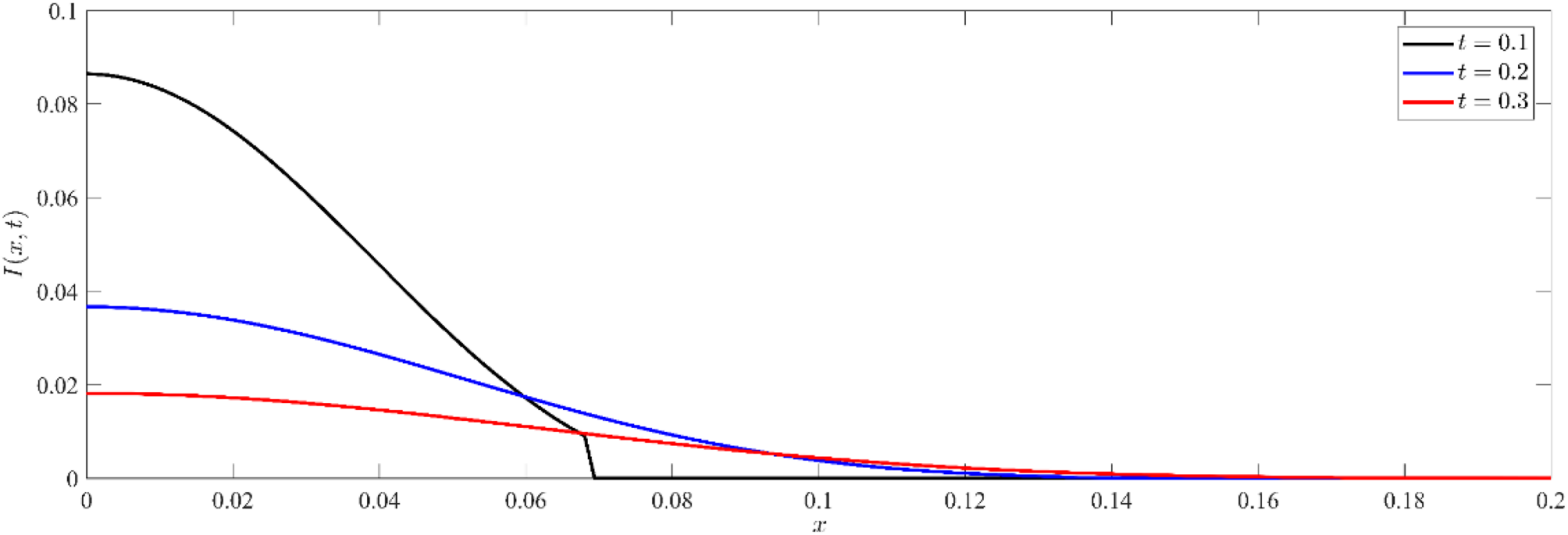
Non dimensional current carrier distribution $I(x,t)$ for different values of time.

Figure 5 shows the non-dimensional distribution of current carriers $I(x,t)$ against $x-$axis for three different instants of time, namely $t=0.1, 0.2$ and $t=0.3$. At the boundary, there is a large value of current carriers on the surface, which decreases as they move into the medium. The initial value represents the prompt reaction of the current carriers to thermal excitation. Over time, the carriers infiltrate more into the silicon, causing a decrease in the original value, which suggests that the carriers are spreading throughout the material.

**Figure 6:**
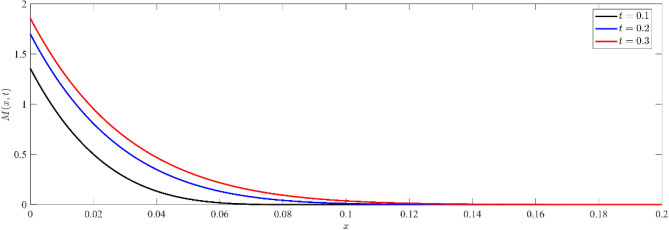
Non dimensional electro-chemical potential energy distribution $M(x,t)$ for different values of time.

Figure 6 illustrates the distribution of non-dimensional electrochemical potential energy $M(x,t)$ for different values of time. The surface of the material has the largest potential energy, which gradually diminishes to zero as one moves deeper into the substance. The observed pattern demonstrates an inverse correlation between the chemical potential and distance.

Based on the Fermi–Dirac distribution, the chemical potential-which stands for the energy required to get an electron into the system-determines the probability that energy levels will be occupied. The chemical potential has a direct impact on the carrier concentration, which consists of holes in the valence band and electrons in the conduction band.

**Figure 7:**
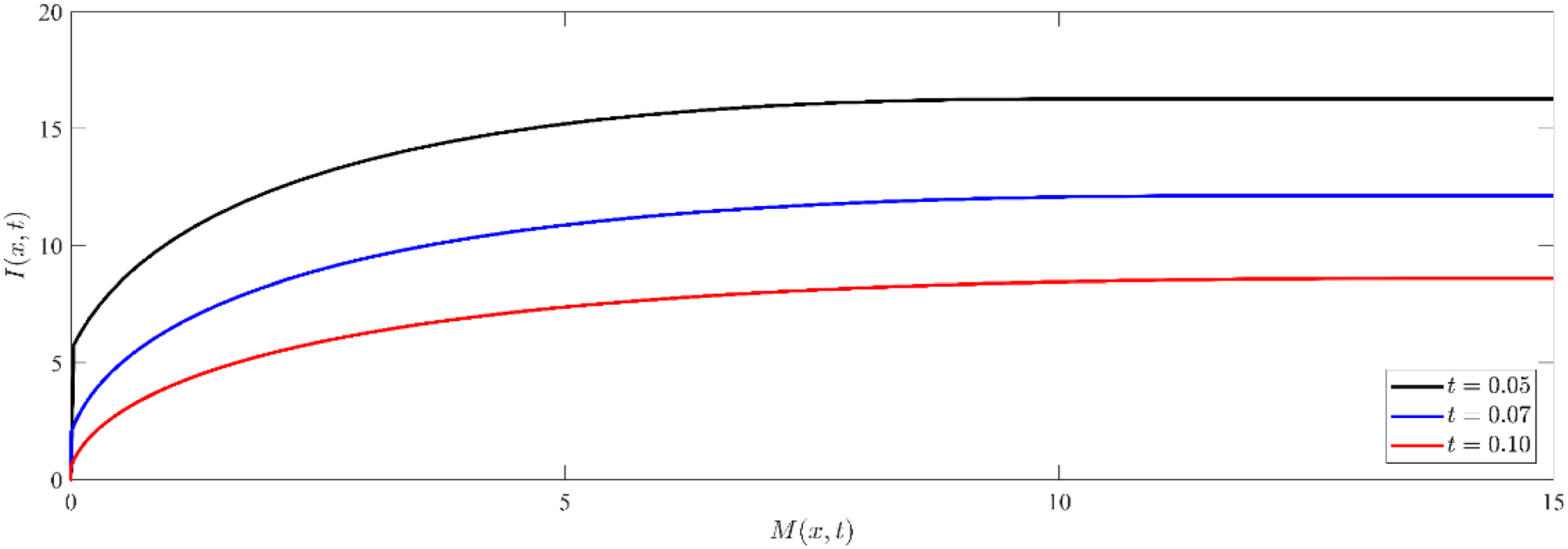
Non dimensional current carrier distribution $I(x,t)$ versus non dimensional electro-chemical potential energy distribution $M(x,t)$ at different values of time.

To check the validation of the results obtained in Figs. 5, and 6, we can represent the relation between chemical potential and the current carriers for different values of time (see, Fig. 7). Figure 7 depicts the correlation between non-dimensional current carriers $I(x,t)$ and electro-chemical potential energy $M(x,t)$ at different values of time in semiconductor materials. The distribution illustrates the interdependence between the current carriers and electrochemical potential, where changes in one entity impact the other^59^.

**Figure 8:**
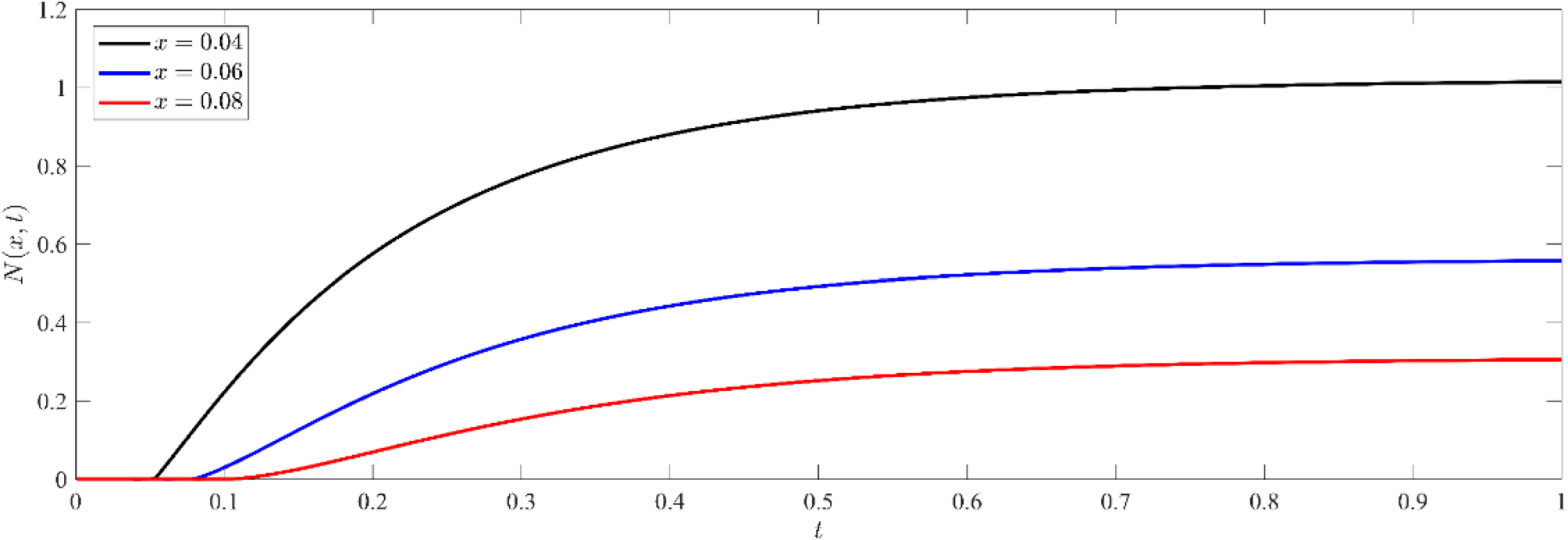
Non dimensional number of particle distribution $N(x,t)$ for different depths inside the medium.

**Figure 9:**
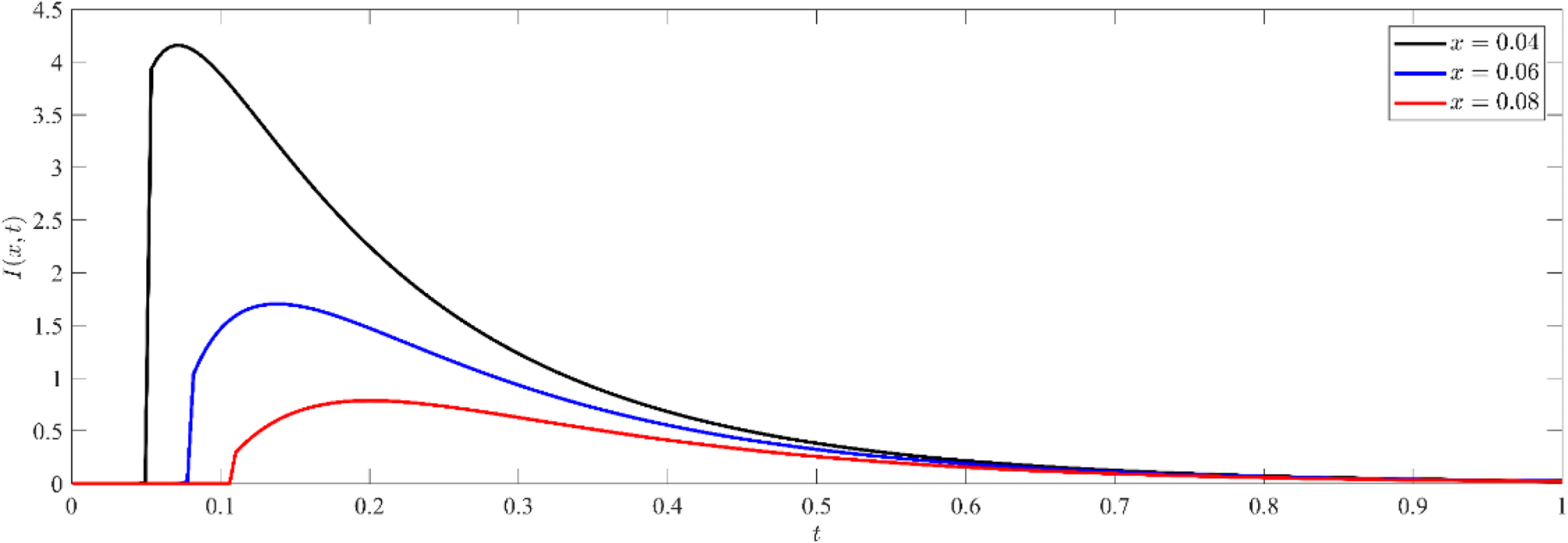
Non dimensional current carrier distribution $I(x,t)$ for different depths inside the medium.

**Figure 10:**
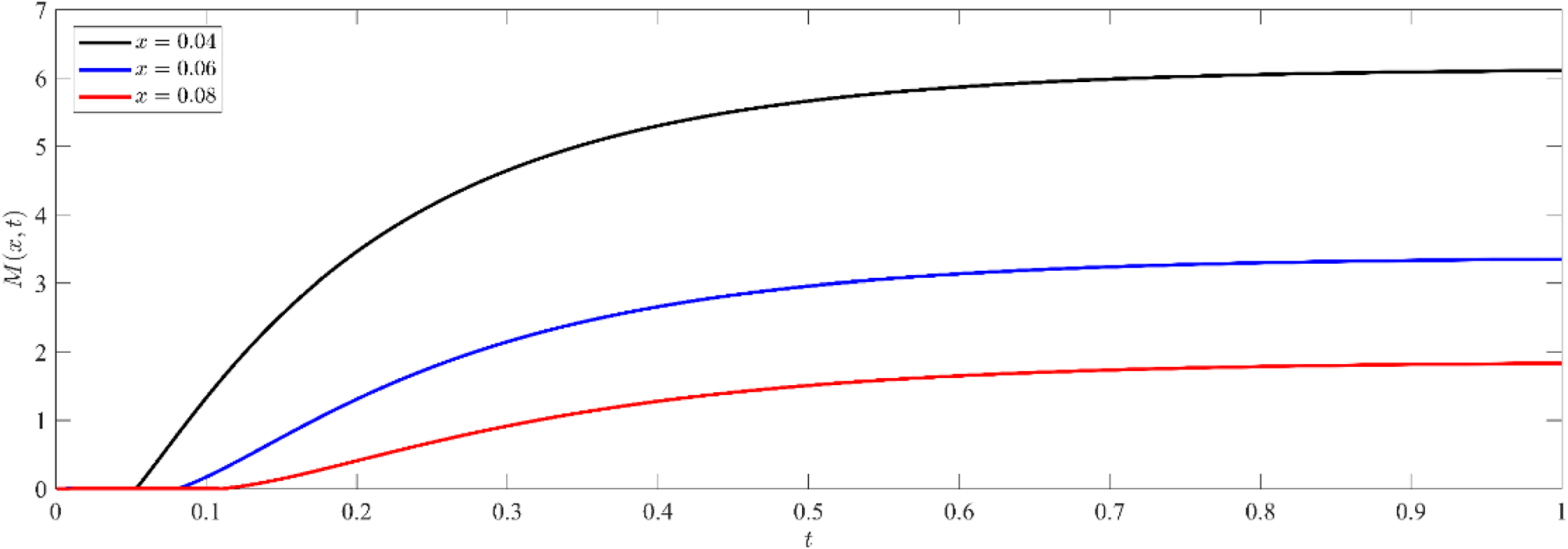
Non dimensional electro-chemical potential energy distribution $M(x,t)$ for different depths inside the medium.

Based on Figs. 8, 9, 10, it is seen that the profile of the exhibited functions begins after a period of delay, during which the wave arrived to this deep inside the medium. As the value of x increases, the wave starts later and propagates farther into the media. This phenomenon is consistent with the physical reality, as the waves penetrate farther into the medium and the wave front eventually reaches a greater depth as the elapsed time grows. Furthermore, the function's value has a greater magnitude for shorter distances (smaller $x$) as opposed to deeper distances. Figure 8 illustrates the non-dimensional current density $N(x,t)$ at various depths within the silicon medium. The concentration of particles decreases as depth increases, indicating the existence of the diffusion process. The phenomenon of wave arrival delay at increasing depths is in accordance with the physical phenomenon of wave propagation. Analogous to the dispersal of particles, the concentration of current carriers diminishes as depth and duration increase, as shown in Fig. 9. By observing Figs. 8, 9, it is evident that the high concentration on the boundary surface diminishes as carriers spread out into the medium by diffusion. This distribution supports the theoretical model of charge carrier dynamics in semiconductors, where thermal stimulation causes a progressive movement of carriers into deeper regions.

Figure 10 demonstrates that the distribution of electrochemical potential energy grows with time at all depths, suggesting a progressive accumulation of electrochemical potential energy inside the medium. As the depth within the medium increases, the electro-chemical potential energy decreases. This behavior can be attributed to diffusion and transport mechanisms, in which the distribution is driven by a gradient of potential energy and eventually reaches a stable state.

### Consent to participate

All authors consent to participate to this publication.

## Particle diffusion relaxation time ${{\varvec{\tau}}}_{1}$: significance and impact on current carrier

In ideal circumstances, the current carriers flowing through semiconductors should have a continuous function. This assumption simplifies theoretical models and circuit analysis, allowing us to apply well-established principles like Ohm's law and Kirchoff's laws. However, under certain practical scenarios, the current carriers could appear discontinuous or contain rapid transitions. Such situations arise from inherent limitations in modeling assumptions, manufacturing imperfections, or complex interactions occurring in advanced electronic systems. So, it was necessary to address the limitations of theoretical models in explaining this phenomenon in our model. Consequently, the modification of Ohm's law, as described in Eq. (34), is needed. To study the effect of this modification according to different values of particle diffusion relaxation time, a Video S1 displays the current carrier distribution $I(x,t)$ inside the medium, which ${\tau }_{1}$ varies from $0$ to $0.02$. We note that for ${\tau }_{1}=0,$ the current carriers behave as a smooth continuous function consistent with the theoretical models. However, for ${\tau }_{1}>0$, the current carriers suffer a sudden jump in their value at the wavefront location, which becomes more noticeable as ${\tau }_{1}$ increases. This covers several experimental situations, where ${\tau }_{1}$ is an arbitrary constant that takes special values according to different physical circumstances.

## Validation of our mathematical model in comparison with many earlier models.

In this section, we will present a generalized mathematical model based on specific mathematical parameters ${\zeta }_{i}, i=\text{1,2},\dots ,8$. We can use this model to derive every earlier model^22,23,60^ as a special case, which are included in Table 2. The proposed governing equations are as follows:

69 $$\mu {u}_{i,jj}+\left(\lambda +\mu \right){u}_{j,ij}-{\beta }_{1}{\theta ,}_{i}-{\beta }_{2}{N,}_{i}+\rho {F}_{i}={\rho \ddot{u}}_{i},$$

70 $$\rho {C}_{E}\left(\dot{\theta }+{\tau }_{0}\ddot{\theta }\right)+{{\zeta }_{1}\beta }_{1}{T}_{0}\left(\dot{e}+{\tau }_{0}\ddot{e}\right)-{\zeta }_{2}{E}_{g}\left(\dot{N}+{\tau }_{0}\ddot{N}\right)-{\zeta }_{3}\frac{{E}_{g}}{{\tau }_{R}}N=k{\theta }_{,ii},$$

71 $$\left(1+{\tau }_{1}\frac{\partial }{\partial \text{t}} \right)\dot{N} +\frac{1}{{\tau }_{R}}\left(1+{\zeta }_{4}{\tau }_{1}\frac{\partial }{\partial \text{t}} \right)N=\frac{{\zeta }_{5}{\sigma }_{0}{E}_{g}}{{q}_{0}^{2}{T}_{0}}{\nabla }^{2}\theta -\frac{{{\zeta }_{6}\sigma }_{0}{\beta }_{2}}{{q}_{0}^{2}} {\nabla }^{2}e+\frac{{\sigma }_{0}b}{{q}_{0}^{2}}{\nabla }^{2}N+\frac{{\zeta }_{7}}{{\tau }_{R}}\frac{\partial {n}_{0}}{\partial T}\theta -{\zeta }_{8}{\gamma }_{u}\left(\dot{e}+{\tau }_{0}\ddot{e}\right),$$

where ${\gamma }_{u}$ is the elastic coupling factor.

**Table 2:** Limiting cases of the suggested model with previous models.

Model		${\zeta }_{i}$	Relaxation times	References
(I)	The presented model	${\zeta }_{1}={\zeta }_{2}={\zeta }_{4}={\zeta }_{5}={\zeta }_{6}=1$	${\tau }_{0}\ne 0, {\tau }_{1}\ne 0$	
(II)	Coupled photo thermal elastic	${\zeta }_{1}={\zeta }_{3}={\zeta }_{7}=1$	${\tau }_{0}={\tau }_{1}=0$	^22,60^
(III)	Generalized photo thermal elastic	${\zeta }_{1}={\zeta }_{7}={\zeta }_{8}=1$	${\tau }_{0}={\tau }_{1}$	^23^

By substituting the non-dimensional variables from the previous Eq. (43), we obtain

72 $${\nabla }^{2}\mathbf{u}+\left({\beta }^{2}-1\right)\nabla e-{\beta }^{2}\nabla \theta -{\beta }^{2}\nabla N={\beta }^{2}\ddot{\mathbf{u}},$$

73 $$\left(\frac{\partial }{\partial t}+{\tau }_{0 }\frac{{\partial }^{2}}{\partial {t}^{2}}\right)\left(\theta +{\zeta }_{1}\varepsilon e-{\zeta }_{2} {\alpha }_{1}N\right)+{\zeta }_{3}{\omega }_{1}N={\nabla }^{2}\theta,$$

74 $${\alpha }_{2}\left[\left(1+{\tau }_{1}\frac{\partial }{\partial \text{t}} \right)\dot{N}+\left(1+{\zeta }_{4}{\tau }_{1}\frac{\partial }{\partial \text{t}} \right)\frac{1}{{\tau }_{R}}N\right]={\zeta }_{5}{\alpha }_{3}{\nabla }^{2}\theta -{\zeta }_{6}{\alpha }_{4} {\nabla }^{2}e+{\alpha }_{5}{\nabla }^{2}N+{\zeta }_{7}{\alpha }_{2}\frac{\partial {n}_{0}}{\partial T}\frac{\theta }{{\tau }_{R}}-{\zeta }_{8}{\alpha }_{8}\left(\dot{e}+{\tau }_{0}\ddot{e}\right),$$

where $${\omega }_{1}=\frac{{E}_{g}{\beta }_{1}}{{\tau }_{R}{C}_{E }{\beta }_{2}\eta \left(\lambda +2\mu \right)}$$, and $${\alpha }_{8}=\frac{{\gamma }_{u}{q}_{0}^{2}}{\eta }.$$

We obtained the solution for the various previous models using the same methods in section "Formulation of the problem", but we don't present them here to avoid expanding the article's body. The Tables 3, 4, 5 are critical for verifying our model's precision. They allow us to compare our model (I) with other models (II, III) by examining non-dimensional temperature, stress and current carrier distributions at various locations ($x$) and two instants of time $(t = 0.05, t = 0.10).$ These comparisons emphasize the model's ability to accurately forecast temperature and stress behavior.

**Table 3:** Non-dimensional temperature distributions at various locations ($x$) and two instants of time $(t = 0.05, t = 0.10).$

Non-dimensional temperature distributions						
$x$	$t=0.05$			$t=0.10$		
	Model (I)	Model (II)	Model (III)	Model (I)	Model (II)	Model (III)
$0.0$	$1.000$	$1.000$	$1.000$	$1.000$	$1.000$	$1.000$
$0.1$	$0.77867$	$0.750107$	$0.750097$	$0.940311$	$0.939975$	$0.939958$
$0.2$	$0.567274$	$0.524103$	$0.524101$	$0.885437$	$0.884807$	$0.884775$
$0.3$	$0.374546$	$0.339385$	$0.339386$	$0.831094$	$0.830195$	$0.830148$
$0.4$	$0.000159$	$0.205373$	$0.205374$	$0.776725$	$0.775587$	$0.775526$
$0.5$	$7.4\times {10}^{-5}$	$0.112976$	$0.112977$	$0.724184$	$0.722858$	$0.722782$
$0.6$	$7.7 \times 10^{ - 6}$	$0.056973$	$0.056974$	$0.667809$	$0.666336$	$0.666246$
$0.7$	$1.4 \times 10^{ - 6}$	$0.026815$	$0.026815$	$0.61809$	$0.61655$	$0.616448$
$0.8$	$1.3 \times 10^{ - 6}$	$0.011321$	$0.011321$	$0.56997$	$0.568406$	$0.568292$
$0.9$	$1.2 \times 10^{ - 6}$	$0.004362$	$0.004362$	$0.523561$	$0.522047$	$0.521921$
$1.0$	$9.1 \times 10^{ - 7}$	$0.001482$	$0.001482$	$0.479159$	$0.477819$	$0.477698$

**Table 4:** Non-dimensional temperature distributions at various locations ($x$) and two instants of time $\left( {t{ } = { }0.05,{ }t{ } = { }0.10} \right).$

Non-dimensional stress distributions						
$x$	$t = 0.05$			$t = 0.10$		
	Model (I)	Model (II)	Model (III)	Model (I)	Model (II)	Model (III)
$0.0$	$0.0$	$0.0$	$0.0$	$0.0$	$0.0$	$0.0$
$0.1$	$- 7.09816$	$- 8.33656$	$- 8.97816$	$0.020102$	$0.04836$	$0.063144$
$0.2$	$- 3.4048$	$- 4.43125$	$- 4.94839$	$0.043858$	$0.09916$	$0.128416$
$0.3$	$- 1.64379$	$- 2.39972$	$- 2.77596$	$0.07441$	$0.153383$	$0.195931$
$0.4$	$- 0.57184$	$- 1.32323$	$- 1.57878$	$0.127696$	$0.229616$	$0.286445$
$0.5$	$- 0.25338$	$- 0.71019$	$- 0.87079$	$0.217438$	$0.336365$	$0.406469$
$0.6$	$- 0.11106$	$- 0.37212$	$- 0.467$	$0.407097$	$0.538833$	$0.622724$
$0.7$	$- 0.04871$	$- 0.19239$	$- 0.24611$	$0.76385$	$0.899316$	$0.995531$
$0.8$	$- 0.02063$	$- 0.09479$	$- 0.12332$	$1.563754$	$1.685266$	$1.787198$
$0.9$	$- 0.00849$	$- 0.04535$	$- 0.05983$	$3.285119$	$3.349079$	$3.420478$
$1.0$	$- 0.00344$	$- 0.02157$	$- 0.02879$	$- 7.14242$	$- 8.91677$	$- 9.58175$

**Table 5:** Non-dimensional temperature distributions at various locations ($x$) and two instants of time $\left( {t{ } = { }0.05,{ }t{ } = { }0.10} \right).$

Non-dimensional current carrier distributions						
$x$	$t = 0.05$			$t = 0.10$		
	Model (I)	Model (II)	Model (III)	Model (I)	Model (II)	Model (III)
$0.0$	$12.79329$	$14.24079$	$14.95495$	$12.81319$	$14.67206$	$15.60121$
$0.1$	$5.812688$	$7.127899$	$7.767796$	$5.721925$	$7.544544$	$8.455605$
$0.2$	$2.636565$	$3.678934$	$4.191972$	$2.555213$	$4.298192$	$5.169461$
$0.3$	$1.192789$	$1.949958$	$2.321085$	$1.208701$	$2.860962$	$3.686878$
$0.4$	$0.546902$	$1.063748$	$1.315375$	$0.539759$	$2.087287$	$2.857984$
$0.5$	$0.244117$	$0.571417$	$0.729561$	$0.255329$	$1.702065$	$2.425302$
$0.6$	$0.107686$	$0.303042$	$0.396491$	$0.114005$	$1.45518$	$2.125541$
$0.7$	$0.047747$	$0.159839$	$0.21281$	$0.053914$	$1.299093$	$1.921379$
$0.8$	$0.020239$	$0.080831$	$0.108989$	$0.02417$	$1.168934$	$1.741051$
$0.9$	$0.008347$	$0.039793$	$0.0541$	$0.010833$	$1.05913$	$1.583121$
$1.0$	$0.003392$	$0.019455$	$0.026586$	$0.005074$	$0.967811$	$1.449053$

**Figure 11:**
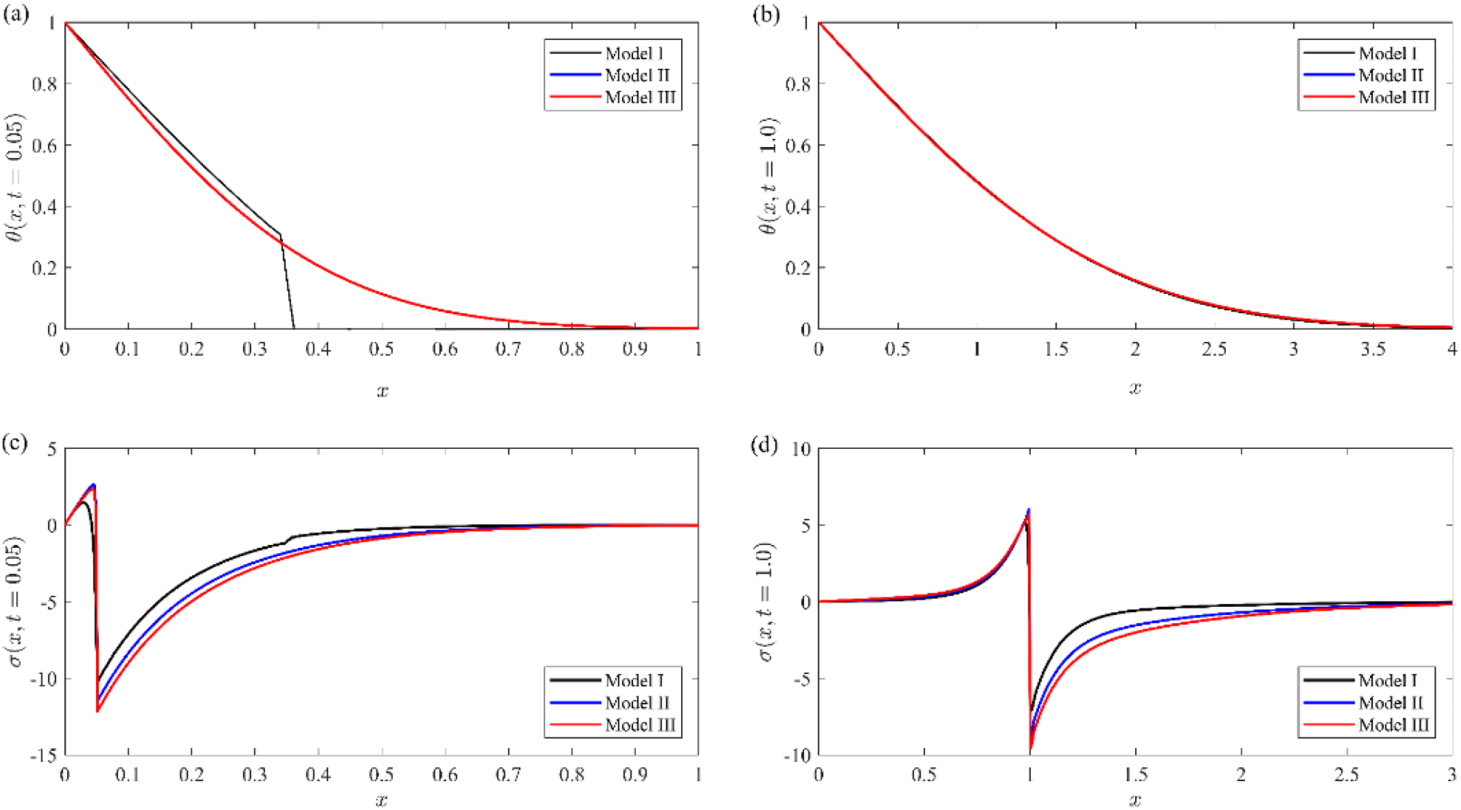
Comparison between the temperature and Normal stress distributions for different models at small and large instant of times.

**Figure 12:**
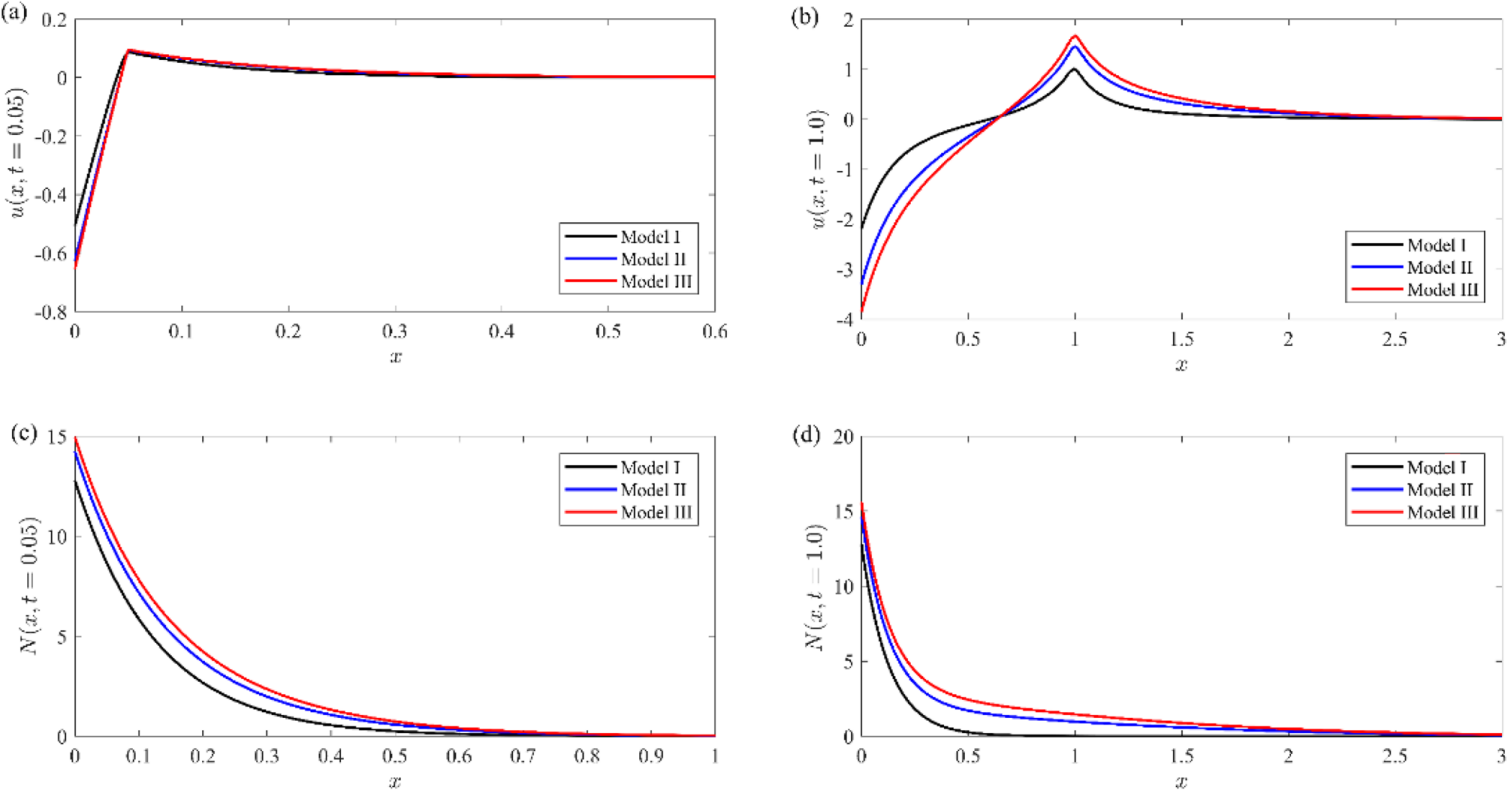
Comparison between the displacement and number of particles distributions for different models at small and large instant of times.

Figures 11, 12 represent a comparison between the above different models (Model I, black line), (Model II, blue line), and (Model III, red line) for non-dimensional physical variable fields, namely, nondimensional temperature distribution (see Fig. 11a, b), nondimensional stress distribution (see Fig. 11c, d), nondimensional displacement (see Fig. 12a, b), and nondimensional number of particles (as in Fig. 12c, d). In those figures, labels (a) and (c) indicate the case of small time ($t = 0.05$), while those labeled by (b) and (d) indicate the case of large time ($t = 1$). The non-dimension relaxation parameters for the generalized model III are taken to be ${\tau }_{0}={\tau }_{1}=0.02$, while those values are ${\tau }_{0}=0.02, {\tau }_{1}=0.01$ for our model I.

From these figures, we note that

(i) The temperature distribution for small values of time in our model has a finite speed, while the others predict an infinite speed, and the wave front location appears at $x = 0.35$. This advantage distinguishes our model, while the other two models behave like the coupled theory of thermoelasticity. Therefore, our model eliminates the paradox in the other two models. The temperature distributions for all models are identical for large values of time.
(ii) The normal stresses component has the same mechanical wave front location for all models at any time, while the thermal wave front location appears only in our model for a short time. There is a slight difference between all models in the tensile region, while it is more prominent in the compressive region.
(iii) The displacement component $u$ distribution has a small change between all models in the case of small time, while there is a prominent change in the case of large time.
(iv) The number of particle distribution $N$ in our model records a smaller value rather than other models and has rapidly decay to zero.
(v) All functions field in our model have a smaller value than the other models and rapid decay in the semiconductor media.

## Conclusion

This study presents an innovative mathematical framework for examining the characteristics of semiconductor elastic materials when subjected to an external magnetic field. The main goal is to provide a comprehensive mathematical model that encompasses the interplay of plasma, thermal, and elastic waves in semiconductor materials. The mathematical modeling of semiconductors now includes the integration of electrochemical potential, marking a significant development. The electrochemical potential in semiconductors is vital as it controls the behavior and concentration of charge carriers, hence directly impacting the performance and functioning of semiconductor devices like diodes and transistors. Furthermore, it establishes the equilibrium conditions and transport phenomena, which are crucial for comprehending and designing semiconductor materials and devices.

### Supplementary Information

Supplementary Information 1.

Supplementary Video 1.

## Data Availability

All data generated or analyzed during this study are included in this published article.

## List of symbols

${\alpha }_{n}$: Discrepancy in deformation potential between the conduction and valence bands
${\alpha }_{t}$: Coefficient of linear thermal expansion
$$\mathbf{B}$$: Magnetic induction
${C}_{ijkl}$: Tensor of elastic constants
${C}_{E}$: Specific heat at constant strain
${D}_{e}$: Carrier diffusion coefficient
$\mathbf{E}$: Applied external electric field
${e}_{ij}$: Components of the strain tensor
$$e={e}_{ii}$$: Cubical dilation
${E}_{g}$: Energy gap of the semiconductor
${F}_{i}$: Components of the external forces per unit mass
$$\mathbf{F}$$: Lorentz force
$G$: Carrier photo generation source per unit charge
${\mathbf{H}}_{0}$: External applied magnetic field
$\mathbf{h}$: Induced magnetic field
$\mathbf{J}$: Current carrier density vector
*k*: Thermal conductivity
$$\lambda$$, $$\mu$$: Lam's constants
*M*: Electro-chemical potential energy
$${\mu }_{0}$$: Magnetic permeability
*n*: Carrier density at time $t$
${n}_{0}$: Carrier density at equilibrium
${n}_{j}$: Components of normal unit vector outward to the surface $A$
$\psi$: Helmholtz free energy function
${q}_{i}$: Heat flux vector
${q}_{0}$: Charge of the particle
*Q*: Intensity of the external heat sources per unit mass
$\rho$: Independent time density of the medium
*S*: Entropy per unit mass
${\sigma }_{ij}$: Components of the stress tensor
$${\sigma }_{0}$$: Electric conductivity
$$T$$: Absolute Temperature
${T}_{0}$: Temperature of the material at equilibrium
${\tau }_{0}$: Thermal relaxation time
${\tau }_{R}$: Electron–hole recombination time
$$t$$: Time
$U$: Internal energy per unit mass
$${u}_{i}$$: Displacement components
