# Supplementary material for: A fully coupled system of generalized thermoelastic theory for semiconductor medium
Source: Sci Rep. 2024 Jun 16;14:13876. doi: 10.1038/s41598-024-63554-2 (PMC11180667; doi:10.1038/s41598-024-63554-2)
Supplement: Supplementary file 1 — Supplementary Information 1. [file 41598_2024_63554_MOESM1_ESM.docx]

**Description of Additional Supplementary File**

File Name: Supplementary Video S1

Description: The effects of particle diffusion relaxation time on the current carrier distribution.

The nondimensional current carrier distribution $I(x,t)$ inside the medium for different particle diffusion relaxation time $\tau_{1}$is displayed. Several experimental situations were covered by the range of the particle diffusion relaxation time $\tau_{1}$ from $0$ to $0.02$
